# Prevalence of onchocerciasis and epilepsy in a Tanzanian region after a prolonged community-directed treatment with ivermectin

**DOI:** 10.1371/journal.pntd.0012470

**Published:** 2024-09-06

**Authors:** Vivian P. Mushi, Dan Bhwana, Isolide S. Massawe, Williams Makunde, Hillary Sebukoto, Willison Ngasa, Joel Sengerema, Athanas Mhina, Paul M. Hayuma, Henrika Kimambo, Winifrida Kidima, William Matuja, Josemir W. Sander, Helen Cross, Arjune Sen, Robert Colebunders, Charles R. Newton, Bruno P. Mmbando, EPInA Study Group

**Affiliations:** 1 Department of Parasitology and Medical Entomology, School of Public Health and Social Sciences, Muhimbili University of Health and Allied Sciences, Dar es Salaam, Tanzania; 2 Department of Zoology and Wildlife Conservation, College of Natural and Applied Sciences, University of Dar es Salaam, Dar es Salaam, Tanzania; 3 Tanga Research Centre, National Institute for Medical Research, Tanga, Tanzania; 4 Tanga Regional Referral Hospital, Tanga, Tanzania; 5 Bumbuli District Council, Tanga, Tanzania; 6 Department of Internal Medicine, Muhimbili National Hospital, Dar es Salaam, Tanzania; 7 Department of Neurology, Muhimbili University of Health and Allied Sciences, Dar es Salaam, Tanzania; 8 Department of Clinical and Experimental Epilepsy, UCL Queen Square Institute of Neurology, London WC1N 3BG & Chalfont Centre for Epilepsy, London, United Kingdom; 9 Neurology Department, West China Hospital, Sichuan University, Chengdu, China; 10 Stichting Epilepsie Instellingen Nederland (SEIN), Heemstede, The Netherlands; 11 Developmental Neurosciences Research and Teaching Department, UCL Great Ormond Street Institute of Child Health, London, United Kingdom; 12 Oxford Epilepsy Research Group, Nuffield Department of Clinical Neurosciences, University of Oxford, Oxford, United Kingdom; 13 Global Health Institute, University of Antwerp, Antwerp, Belgium; 14 Neuroscience Unit, KEMRI-Wellcome Trust Research Programme, Kilifi, Kenya; 15 Department of Psychiatry, University of Oxford, Oxford, United Kingdom; Chung-Ang University, REPUBLIC OF KOREA

## Abstract

**Introduction:**

Epidemiological evidence suggests that *Onchocerca volvulus* is associated with epilepsy, although the exact pathophysiological mechanism is unknown. Mahenge is an endemic focus of onchocerciasis, with the longest-running ivermectin treatment intervention in Tanzania. We assessed the prevalence of onchocerciasis and epilepsy after 25 years of control using ivermectin.

**Methods:**

This was a population-based cross-sectional study in 34 villages in Mahenge in 2021. Community health workers conducted door-to-door household surveys to enumerate the population and screen for individuals suspected of epilepsy using a standardised questionnaire. Trained physicians confirmed epilepsy. Children aged 6–11 years were screened for onchocerciasis antibodies using the Ov16 rapid test. Villages were stratified into three altitude levels (low [<400], medium [400–950], and high [>950 meters above sea level]) as a proxy for rapids, which black flies favour for breeding sites. Incidence of epilepsy was estimated as a ratio of new cases in the year preceding the survey per 100,000 population.

**Results:**

56,604 individuals (median age 20.2 years, 51.1% females) were surveyed. Onchocerciasis prevalence in children was 11.8% and was highest in villages at medium (21.7%) and lowest in low altitudes (3.2%), p<0.001. Self-reported use of ivermectin was 88.4%. Epilepsy prevalence was 21.1 (95%CI: 19.9–22.3) cases per 1000 persons and was highest in medium (29.5%) and lowest in the lowlands (12.7%). The odds ratio (OR) of having epilepsy was significantly higher in females (OR = 1.22, 95%CI: 1.08–1.38), middle altitudes (OR = 2.34 [95%CI: 2.04–2.68]), and in individuals positive for OV16 (OR = 1.98 [95%CI:1.57–2.50]). The incidence of epilepsy a year before the survey was 117 (95%CI: 99.7–160.4) cases per 100,000 person-years.

**Conclusion:**

Despite ivermectin use for 25 years, the prevalence of onchocerciasis and epilepsy remains high. It is crucial to strengthen bi-annual ivermectin treatment and initiate interventions targeting vectors to control onchocerciasis and epilepsy in the area.

## Author summary

Onchocerciasis is endemic in Mahenge, with epidemiological evidence that the parasite (*Onchocerca volvulus*) may be associated with epilepsy. The high burden of the disease in Mahenge led to the introduction of Community Directed Treatment with Ivermectin (CDTI) in 1997. Despite the long history of CDTI implementation in Mahenge, the persistent transmission of the parasite is still reported. A population-based cross-sectional study was conducted to establish the current prevalence of onchocerciasis and epilepsy 25 years after the start of the CDTI programme.

The prevalence of epilepsy was 21.1 (95%CI: 19.9–22.3) cases per 1000 people. The incidence of epilepsy one year preceding the survey was 117 (95%CI: 99.7–160.4) per 100,000 person-years. Despite high self-reported coverage of ivermectin (88%), active onchocerciasis transmission was observed among children aged 6–11 years, and more than half of the individuals with epilepsy (54.1%) were serologically positive for onchocerciasis. The predictors for epilepsy were sex (female), age ≥ 10 years, being a resident of Mahenge for ≥20 years, especially in villages located at a medium altitude, and being onchocerciasis positive. Strengthening of processes to ensure bi-annual ivermectin treatment is recommended.

## Introduction

Onchocerciasis, a parasitic disease due to *Onchocerca volvulus* (*O*. *volvulus*), causes significant morbidity and mortality in endemic areas [1]. The disease vectors are infected black flies (*Simulium* spp), which reproduce in fast-flowing water, thus this disease is called river blindness [2]. Onchocerciasis is responsible for skin (lesions, onchodermatitis) and ocular (conjunctivitis, sclerosing, keratitis, and blindness) manifestations [3]. Over 200 million people are at risk of onchocerciasis, with almost all cases occurring in sub-Saharan Africa. It is estimated that more than 40 million people are currently infected [2,4].

The high disease burden led to the introducing of control interventions such as community-directed treatment with ivermectin (CDTI) and vector control with environmentally safe methods [2]. CDTI is a core strategy recommended by the WHO and was predicted to control the infestation within 16–18 years at coverage of at least 80% of the population [5]. In some endemic areas, CDTI deployment has significantly reduced onchocerciasis transmission [6,7]. In other regions, despite decades of CDTI, there are still persistent onchocerciasis transmission [8,9] and onchocerciasis-associated morbidities such as epilepsy [10–12].

A high prevalence of epilepsy has been reported in onchocerciasis endemic areas. Recent epidemiological evidence from sub-Saharan Africa suggests that *O*. *volvulus* is associated with epilepsy, and is referred to as onchocerciasis-associated epilepsy (OAE) [11–15]. Some suggest that nodding syndrome, characterised by head nodding attacks, cognitive deterioration, and stunted growth, coupled with other presentations such as Nakalanga syndrome (features include delayed puberty, mental impairment, and stunted growth), are forms of OAE [16]. The exact pathophysiological mechanism by which *O*. *volvulus* is associated with epilepsy remains unknown.

In Tanzania, onchocerciasis is endemic, with more than 6.5 million people at risk [17]. The prevalence of onchocerciasis varies across the country, with the Mahenge area having the highest regional prevalence since the 1960s [17]. The annual CDTI programme in Tanzania started in Mahenge in 1997 [12]. Two decades later, onchocerciasis transmission was still active, with some areas reporting a prevalence of high seropositivity, up to 40% amongst the rural population [11]. Low CDTI uptake and adherence, suboptimal programme implementation, and other challenges are considered responsible for this high prevalence [18]. Persistent transmission is regarded as the main reason for the continued high incidence and prevalence of epilepsy in Mahenge [10,11,19].

An OAE incidence rate of 131 cases per 100,000 person-years was reported in four villages (two peri-urban and two rural villages) in 2018, with rural areas having the highest rates [11]. As a result, in 2019, the Tanzania Neglected Tropical Diseases Control Programme (TNTDCP) initiated bi-annual ivermectin treatment in Mahenge. Current data on the burden of onchocerciasis and epilepsy in Mahenge following this supervised administration of ivermectin is limited. We assessed the current burden of onchocerciasis and epilepsy in the Mahenge area following the long-term use of ivermectin (25 years) for onchocerciasis control. A principal aim was to provide national agencies with an update on the current status of onchocerciasis transmission and associated morbidities. This will enable data-driven programmatic changes to accelerate onchocerciasis control and help the country to eliminate onchocerciasis.

## Materials and methods

### Ethics statement

Ethical approval was provided by the National Institute of Medical Research Ethics Committee (NIMR/HQ/R.8a/Vol.IX/3582). Permission to conduct the study in the Mahenge area was obtained from the appropriate administrative units. All participants were given information about the study before being asked to provide written consent. Only consenting individuals were enrolled. Adults (≥18 years) provided their informed consent by signing the informed consent. For children and minors, permission was sought from their parents or guardians, who signed the consent forms. Additionally, children aged 12–17 years were asked for verbal assent to be involved in the study.

### Study area and demographics

This study was carried out in Ulanga district, one of the seven districts of the Morogoro region. The district lies at latitude 8° 40’ 59" S and longitude 36° 43’ 0" E and is bordered to the east by the Lindi region, to the west and north by the Kilombero district, and south by the Ruvuma Region. The district has an area of 24,460 km^2^ divided into four divisions, 21 wards, and 59 administrative villages with an approximate population of 265,000 [20]. Thirty-four villages ranging from 306 to 1200 meters above sea level (mASL) were involved.

The district experiences an annual rainfall of 1200mm to 1800mm, and temperatures range from 22°C to 33°C. The Mahenge area is mountainous, with more than 40 fast-flowing rivers and streams providing breeding habitats and supporting *Simulium damnosum s*.*I* survival (vector in the district).

### Study design and population

A population-based cross-sectional study was conducted between August and November 2021. All households and household members living in selected villages were eligible to participate.

### Selection of the study villages

Initially, a brief health facility-based survey was implemented in health facilities in the Malinyi and Ulanga districts to assess hospital attendance. Implemented between May and June 2021, this survey aimed to estimate the number of people with epilepsy in the catchment area. A list containing names of wards, villages, and population projections for 2018, 2019, and 2020 was obtained from the district executive office in the Ulanga district. Village visits were organised concurrently to collect information from village executive officers to complement the health facility data. Community health workers (CHWs) in villages provided information on the number of people with epilepsy as they are more conversant with health information in their settings. Where data was missing, CHWs were requested to liaise with community members to ascertain the number of people with epilepsy. The prevalence of epilepsy was estimated as the number of epilepsy cases obtained from health facilities or village executive officers per 1000 population. Villages with a high estimated prevalence of epilepsy were selected purposively to be involved.

### Epilepsy screening and assessment of ivermectin coverage

A two-step approach was used to identify people with epilepsy. A five-question validated epilepsy screening questionnaire was used in the door-to-door household survey [21]. Trained CHWs embedded in the study villages administered the questionnaire to all individuals in a household **(S1 Text)**. Those with at least one positive answer to the questions (screen positive) were invited to a central point for clinical evaluation and diagnostic confirmation by a trained physician (DB, WM, HS, WN, and JS) (**S2 Text**). Individuals diagnosed with epilepsy were also tested for *O*. *volvulus* antibody detection using the Ov16 rapid test. People who failed to attend the central point were traced back to their households for diagnostic confirmation. Those newly confirmed to have epilepsy were referred to a nearby health facility for care. Participants were interviewed about their history of ivermectin use. Answers from children were confirmed by their parents or legal guardians.

### Determining onchocerciasis active transmission

The WHO recommends testing children aged 6–10 years to monitor the active transmission of *O*. *volvulus* in the community under CDTI. The test identifies the existence of active transmission in the community but cannot differentiate between past exposure and active transmission. In children aged 6–11, a finger prick blood sample was obtained for *O*. *volvulus* antibody detection using the Ov16 antigen rapid test (Standard Diagnostics, Incorporated, Gyeonggi-do, Republic of Korea).

### Outcome and independent variables

The study outcomes were the proportion of people who tested positive for onchocerciasis using *O*. *volvulus* IgG4 antibodies detected by Ov16 rapid tests and those confirmed to have epilepsy according to accepted criteria. Independent variables included sex (female or male), age groups, residential village, duration of residence in the study area, altitude (306–1200 meters above sea level), family size, and history of recent use of ivermectin.

### Data management and analysis

A database was created and uploaded using the open-source software Open Data Kit (ODK) [https://opendatakit.org/]. Data were collected using tablets and uploaded daily to a server, where the data was checked for consistency. CHWs resolved queries raised. The ODK data was exported to STATA version 17 (Stata Corp Inc., TX, USA) and R. version 4.0.3 (R Core Team 2020, Vienna, Austria) for analysis. Descriptive statistics were used to summarise the categorical variables into counts/frequency, while continuous variables, median (with interquartile range [IQR]), and/or means with standard deviations were used. The onchocerciasis seroprevalence, prevalence of epilepsy, and ivermectin uptake were further summarised based on the villages and by altitude. To reduce the bias in underreporting epilepsy due to non-response for various reasons, we assumed a proportion of confirmation to be similar between those who responded and the non-responders. Participants were stratified into different age categories. Altitude was grouped into three levels (low, medium, and high) as a proxy for rapid water flows, favouring breeding sites for *Simulium* spp.

The incidence of epilepsy was estimated as the number of epilepsy cases in which seizures started in the previous year, divided by the population size for the period. A one-year period is likely to have less recall bias but may be affected by underreporting due to stigma. We also estimated the incidence based on the five years preceding the study, which is more robust in estimating uncertainty around the mean due to more data in the numerator. We assumed a uniform population across the five years.

Proportions were compared using a χ^2^-test, while means were compared using a t-test. Univariate logistic regression was used to assess the association between explanatory variables and epilepsy, whereas a multivariate model was used to control for confounding variables. All independent variables with p-value <0.1 were included in the multivariate model to adjust for the confounding variables. The findings were reported as adjusted odds ratios (aOR), and a p-value < 0.05 was deemed significant.

### Definitions of key terms

Epilepsy cases were defined and confirmed according to the older classification of ILAE as the presence of two or more unprovoked seizures more than 24 hours apart due to the unavailability of brain imaging or electroencephalography [22]. This allows for meaningful comparisons with previous results in Mahenge and minimises the variability introduced by changes in diagnostic criteria. A household was defined as a person or people living together and eating from the same cooking pot [11]. An onchocerciasis-positive case was defined as someone who tested positive for *O*. *volvulus* IgG4 antibodies using Ov16 tests.

## Results

### Baseline characteristics of the study participants

During the house-to-house survey, 14,644 households were visited comprising 56,604 individuals. More than half of the participants were females (28,895, 51.1%), with the median age of participants being 20.2 years (inter-quartile range (IQR): 9.3–39.1) (Table 1).

**Table 1:** Demographic characteristics of the study population by confirmed epilepsy status.

Characteristic	Population size	Number without epilepsy	Number with epilepsy, n (%)	Test statics, P-value
**Number of households,** n	14,644	13,581 (92.7)	1063 (7.3)	
**Population**	56,604	55,463 (98.0)	1141 (2.0)	
**Age (years), median (IQR**)	20.2 (9.3–39.1)	20.1 (9.3–39.1)	27.8 (17.2–39.0)	χ^2^ = 183, p< 0.001
**Sex**				
Males (%)	27,709 (48.9)	27,173 (98.1)	536 (1.9)	χ^2^ = 8.1, p = 0.005
Females (%)	28,895 (51.1)	28,237 (97.7)	658 (2.3)	
**Age group (years), n (%)**				
0–9	14,935	14,786 (99.0)	149 (1.0)	χ^2^_(6)_ = 290, p < 0.001
10–19	13,079	12,868 (98.4)	211 (1.6)	
20–29	8,192	7,891 (96.3)	301 (3.7)	
30–39	6,753	6,502 (96.3)	251 (3.7)	
40–49	5,352	5,229 (97.7)	123 (2.3)	
50–59	3,734	3,662 (98.1)	72 (1.9)	
60+	4,471	4,388 (98.1)	83 (1.9)	
Missing	88	84 (95.4)	4 (4.6)	
**Altitude (meters above sea level)**				
Low (<400m)	20,860	20,527(98.4)	333 (1.6)	χ^2^_(2)_ = 159, p< 0.001
Medium (400-950m)	21,680	21,010 (96.9)	670 (3.1)	
High (>950m)	14,064	13,873 (98.6)	188 (1.4)	
**Ivermectin use in Aug 2018 for ≥ 5 years**				
Yes (%)	42,491 (88.4)	41,558 (88.4)	933 (86.9)	χ^2^ = 2.4, p = 0.123
No (%)	5,596 (11.6)	5,455 (11.6)	141 (13.1)	

**Fig 1:**
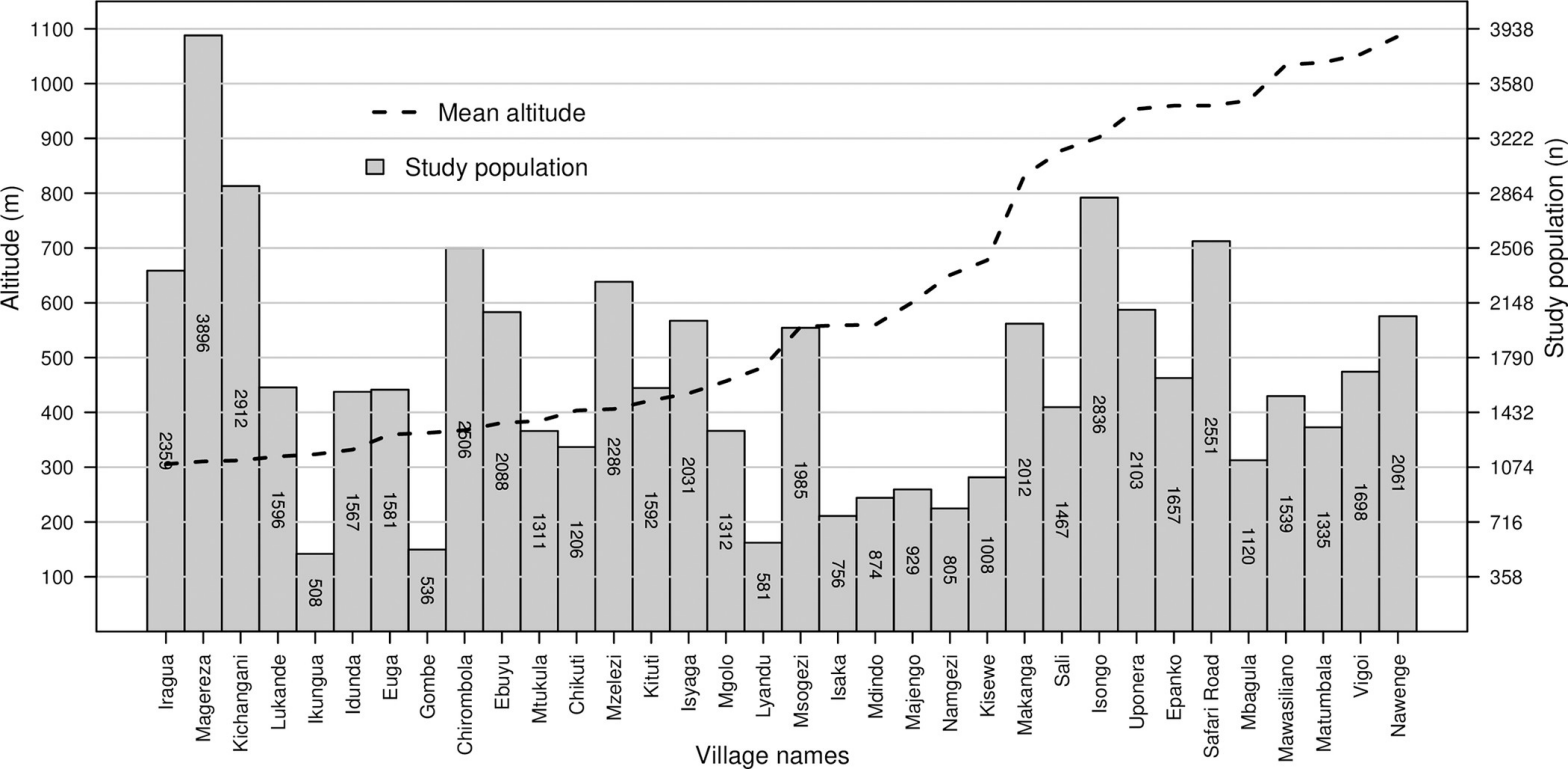
Summary of study villages’ population and altitude. The bars indicate the enumerated study population size, while the dashed line shows the median altitude of the study villages.

The study area included villages with median altitudes ranging from 306 to 1086 meters above sea level. Magereza village had the highest population (3,896 people), while Ikungua village had the lowest (508 people), **Fig 1**.

### Prevalence of epilepsy among the study participants

**Fig 2:**
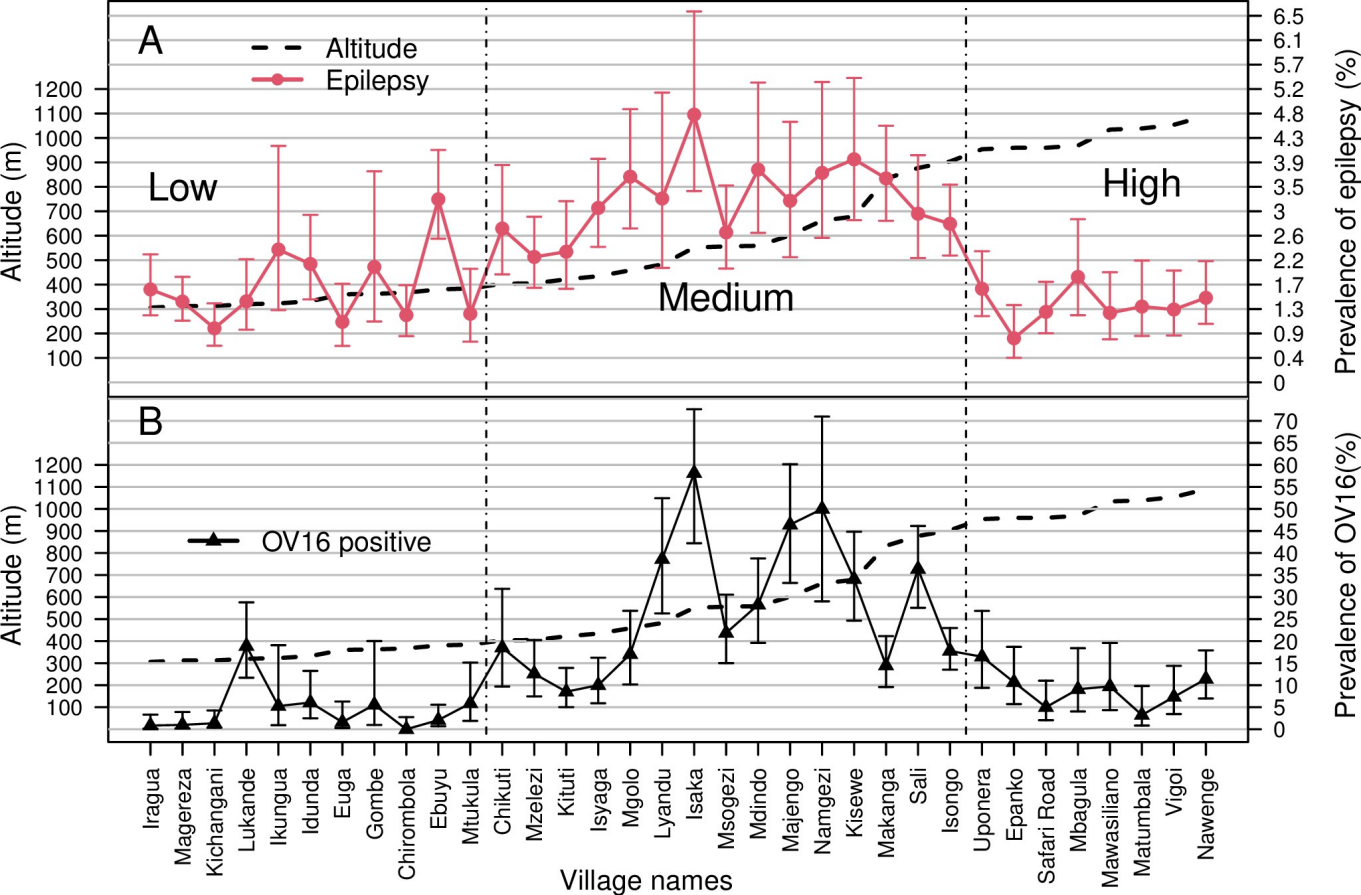
Prevalence of confirmed epilepsy and onchocerciasis by village and altitude (low, medium, and high altitudes). Panel A shows the prevalence of epilepsy (red line), while the dotted line is the median altitude. Panel B shows the prevalence of Ov16 positivity (solid line), while the median altitude is plotted using a dotted line.

Fig 2 displays the prevalence of epilepsy and Ov16 test findings by village and altitude in the study population. A total of 1946 (3.4%) individuals were suspected of having epilepsy, of whom 155 (8.0%) were not available for confirmation of epilepsy while 1194 were confirmed to have epilepsy, giving an overall prevalence of 21.1 (95%CI: 19.9–22.3) cases per 1000 persons. Adjust for participants who did not show up for confirmation, assuming a similar proportion of confirmation (61 for 100 suspected), and the prevalence of epilepsy was 22.8 cases per 1000 (95%CI, 21.6–24.0).

Based on confirmed epilepsy, the villages between medium altitudes of 400–950 meters above sea level had a significantly higher prevalence of 3.1% (95%CI: 2.9–3.3) compared to villages at low and high altitudes (Table 1). Isaka village, at a medium altitude, had the highest prevalence (4.8%), while villages with the lowest prevalence in the low and high altitudes were Kichangani (1.0%) and Epanko (0.8%; **Fig 2**).

**Fig 3:**
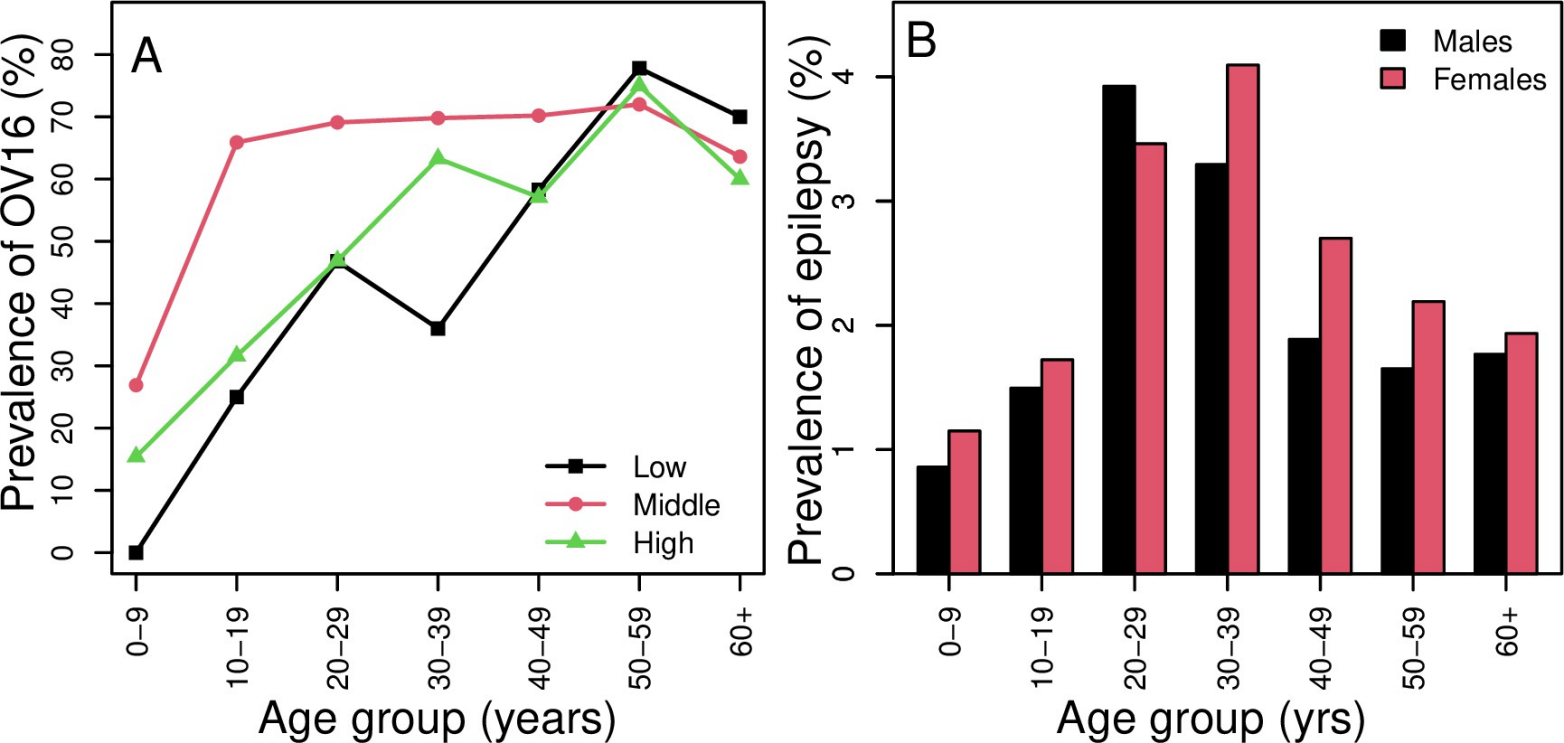
Prevalence of Ov16 positivity by age group (A) among participants with confirmed epilepsy by strata for low (full line), medium (dashed line), and high altitudes (dotted line). Panel B shows the prevalence of epilepsy by age group for males (black bars) and females (red bars).

The prevalence of epilepsy was significantly higher in females (2.3, 95%CI: 2.1–2.5%) than in males (1.9%, 95%CI: 1.8–2.1), p = 0.005. In addition, a more significant epilepsy burden was seen in the age groups of 20–39 years (3.7%), with females aged 30–39 years having the highest prevalence (**Fig 3)**.

### Ov16 seroprevalence among individuals with epilepsy

The overall prevalence of Ov16 positivity among people with epilepsy was 54.1% (474/876) and was highest in villages located at medium altitudes (64.2%), followed by villages at high altitudes (49.6%) and lower laying villages (36.6%), χ^2^ (3) = 52.2, p<0.001.

Ov16 seroprevalence in people with epilepsy increased with age in the entire study area. In the low and high altitudes, seropositivity rose from 0% and 15.4% in the group aged 0–9 years to a peak (>65%) in those aged 50–59. In middle altitudes, the prevalence increased from 25.5% in the age group 0–9 years to >65% in the age group 10–19 years, suggesting the risk of infection is higher in this altitude band (**Fig 3A**).

The prevalence of epilepsy increased with age, reaching its maximum in people aged 20–39 years. It was also observed that, except for 20–29 years, the prevalence was higher among females than males (**Fig 3B**).

### Ov16 seroprevalence and risk factors among children aged 6–11 years

Ov16 seroprevalence amongst the children in this age range was 11.8% (n = 3731, 95%CI: 10.8–12.9), with a high prevalence observed among the children from Isaka village (57.4%). By contrast, none of the children from Chirombola were infected (Fig 2).

Ov16 seroprevalence was highest in the villages located at medium altitudes (21.7%), followed by highlands (9.3%), and lowest in the lowlands (3.2%; p<0.001). The logistic regression model shows the risk (odds ratio) of being Ov16 positive was 8.45 times (95%CI: 6.16–11.61, p<0.001) in children living at medium altitudes. For those in the highlands, it was 3.17 (95%CI: 2.17–4.62, p<0.001) when compared to children living in the lowlands (Table 2).

**Table 2:** Risk factors for Ov16 positivity among the study participants (age 6–11 years).

Variable	Univariate analysis		Multivariate analysis	
	OR (95%CI)	P-value	aOR (95%CI)	P-value
**Sex**				
Males	1			
Females	0.95 (0.78–1.16)	0.615		
**Age** (years)	1.26 (1.17–1.35)	<0.001	1.27 (1.18–1.36)	<0.001
**Duration of residence** (years)	1.12 (1.06–1.19)	<0.001		
**Family size** (people)	1.04 (0.99–1.09)	0.160		
**Ivermectin uptake**				
Yes	1			
No	1.01 (0.67–1.50)	<0.972		
**Altitude** (meters above sea level)				
Low (<400m)	1		1	
Medium (400-950m)	8.35 (6.09–11.46)	<0.001	8.45 (6.16–11.61)	<0.001
High (>950m)	3.10 (2.13–4.52)	<0.001	3.17 (2.17–4.62)	<0.001

*sOR*: Crude Odds Ratios, *aOR*: Adjusted Odds Ratios (age, ivermectin uptake and altitude)_

**Fig 4:**
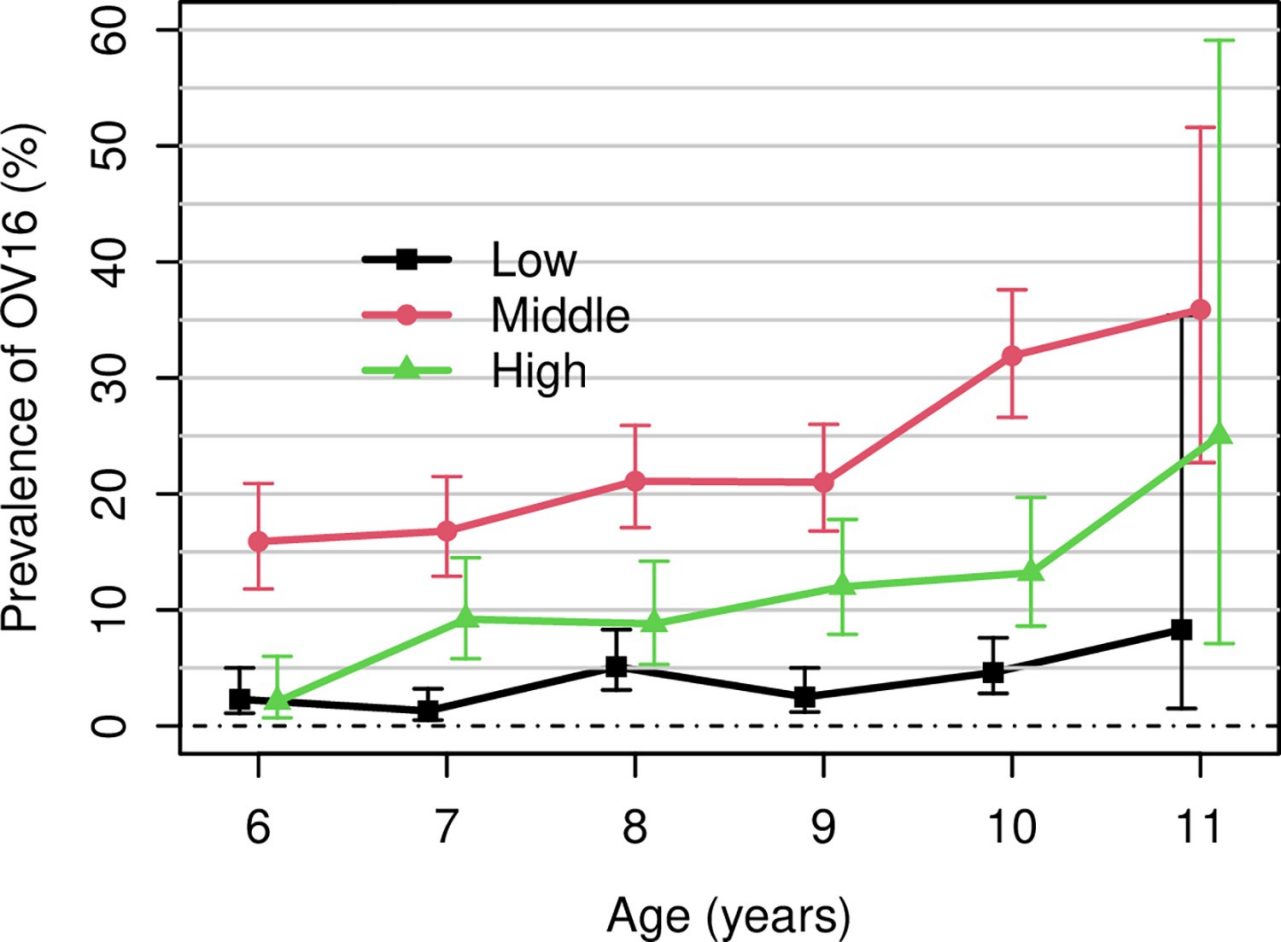
Prevalence of Ov16 positivity with associated 95%CI amongst children aged 6–11 years by altitude strata. The full line represents low altitudes; the dashed line represents middle altitudes; the dotted line represents high altitudes. The error bars are plotted to avoid overlay between the altitudes strata.

Most children had received their first dosage of ivermectin by the age of six years, but more than 15 per cent of children of this age range from communities located at medium altitudes tested positive for Ov16, with the highest prevalence observed among children aged 11 years (**Fig 4**).

### Self-reported uptake of ivermectin among the study participants

The self-reported uptake of ivermectin in the last round of CDTI was 88.4% (42,492/48,087) among the study participants aged ≥ 5 years, with the highest uptake recorded in Chirombola (94.3%) and Isyaga (94.1%) villages. The lowest uptake recorded was in Magereza (67.8%), (S1 Table).

Among the participants aged ≥ 5 years and screened for onchocerciasis, those who did not take ivermectin in the last round had a higher prevalence of Ov16 compared to those who took ivermectin (24.4% vs. 20.0%, χ^2^ = 4.4, p = 0.037). There was no association between the history of use of ivermectin and epilepsy (χ^2^ = 2.4, p = 0.123), neither correlation between village coverage of the ivermectin and prevalence of epilepsy (Pearson correlation = 0.09, 95%CI: (-0.28–0.44), p = 0.636

### Predictors for epilepsy among the study participants

Table 3 illustrates results from univariate and multivariate models showing the association between epilepsy and explanatory variables. In the multivariate model, Ov16 status was omitted since less than 10% of the participants (majority 6–10 years) were screened for onchocerciasis; instead, reported the effect of the Ov16 test is based on a multivariate model fitted for 4464 records.

**Table 3:** Predictors of epilepsy among the study participants.

Variable	Univariate analysis		Multivariate analysis (n = 49,992)	
	OR (95%CI)	P-value	aOR (95%CI)	P-value
**Sex**				
Males	1		1	
Females	1.18 (1.05–1.33)	0.005	1.22 (1.08–1.38)	0.002
**Age (years)**				
0–9	1		1	
10–19	1.63 (1.32–2.01)	<0.001	1.60 (1.28–1.99)	<0.001
20–29	3.79 (3.10–4.62)	<0.001	2.30 (1.79–2.96)	<0.001
30–39	3.83 (3.12–4.70)	<0.001	2.27 (1.75–2.96)	<0.001
40–49	2.33 (1.83–2.97)	<0.001	1.42 (1.02–1.96)	0.036
50–59	1.95 (1.47–2.59)	<0.001	1.21 (0.83–1.76)	0.329
60+	1.88 (1.43–2.46)	<0.001	1.20 (0.82–1.75)	0.352
**Duration of residence (years)**				
0–19	1		1	
20–39	2.89 (2.53–3.29)	<0.001	1.91 (1.57–2.32)	<0.001
40+	1.46 (1.23–1.74)	<0.001	1.59 (1.17–2.16)	0.003
**Family size (people)**				
≤5	1			
6–11	0.80 (0.70–0.90)	<0.001		
≥12	1.11 (0.68–1.84)	0.671		
**Ivermectin uptake (≥ 5 years)**				
Yes	1			
No	1.16 (0.96–1.41)	0.113		
**Altitude (mASL)**				
Low (<400m)	1		1	
Medium (400-950m)	1.97 (1.72–2.24)	<0.001	2.34 (2.04–2.68)	<0.001
High (>950m)	0.85 (0.71–1.02)	0.001	0.82 (0.68–0.98)	0.029
*OV16 tests status				
Negative	1		1	
Positive	6.81 (5.81–7.98)	<0.001	1.98 (1.57–2.50)	<0.001

*OR*: Crude Odds Ratios, *aOR*: Adjusted Odds Ratios, *Based on a multivariate model with 4,464 records and pseudo R^2^ = 37.4%

Estimates from univariate and multivariate models (49,992 records; R^2^ = 4%) were similar, with slight variations by age group. The low score indicates that only about 4% of the observed variations in the prevalence could be explained by variables included in the model. Factors significantly associated with developing epilepsy in the multivariate model were: sex, age (by group), duration of residency, altitude, and onchocerciasis status (Table 3). The multivariate model (4464 records) suggests that people with positive Ov16 results had a risk of having epilepsy (aOR) of 1.98 (95%CI: 1.57–2.50), p<0.001).

### Incidence of epilepsy in Mahenge

Sixty-six participants developed epilepsy in the year preceding the survey, giving an incidence of 117 (95%CI: 90–148) per 100,000 person-years. Residents of villages in the middle altitude had the highest incidence: 184 (95%CI: 131–251) cases per 100,000 person-years, 96 (95%CI: 59–148) in the lowland, while in the highlands it was 42 (95%CI: 17–93) cases per 100,000 person-years. If we adjust for participants who did not have confirmation, the epilepsy incidence was projected at 127 cases per 100,000 (95%CI, 99–160).

**Table 4:** Results from multivariable Poisson regression model showing incidence rate ratios (IRR).

Variable	IRR	95%CI	P-value
**Altitude (mASL)**			
Low (<400m)	1		
Medium (400-950m)	1.39	1.06–182	0.017
High (>950m)	0.60	0.41–0.88	0.009
**Age (years)**			
0–9	1		
10–19	1.05	0.79–1.39	0.752
20–29	0.69	0.47–1.01	0.055
30–39	0.47	0.30–0.75	0.001
40–49	0.23	0.11–0.46	<0.001
50–59	0.32	0.15–0.65	0.002
60+	0.23	0.11–0.50	<0.001
**Sex** Males	1	-	
Females	1.32	1.04–1.69	0.023
Cons (per 100,000)	83.0	52.2–131.9	<0.001

Cons: estimates baseline incidence rate, IRR: Incidence rate

**Fig 5:**
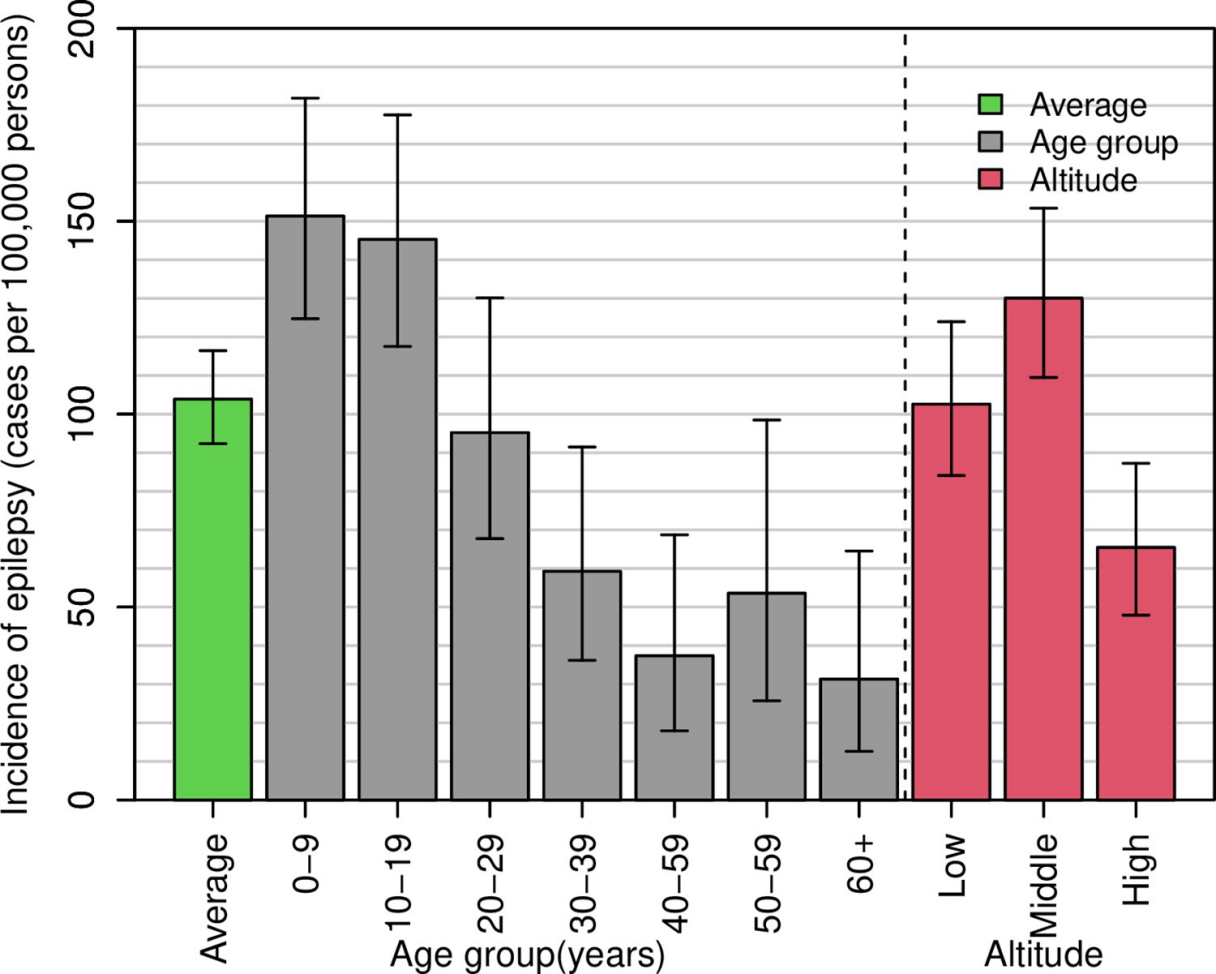
Incidence of confirmed new epilepsy cases based on five years: Average incidence is indicated by the green bar. Grey bars show incidence by age group and the red bars illustrate incidence separated by village altitude.

The five-year incidence was slightly lower than that based on one year: 96 (95%CI: 85–108) per 100,000 person-years. Table 4 shows factors associated with epilepsy incidence over the past five years. Compared to the lowlands, the incidence of epilepsy was 39% higher in the medium-altitude villages and 40% lower in the highlands. Incidence was highest in young children (individuals aged 10 to 19 years) and decreased with age, while females had a significantly higher incidence of 32% compared to males (p = 0.023). The trend of confirmed epilepsy incidence based on five years by altitude strata and age group is presented (**Fig 5**).

## Discussion

We present epidemiological data on the current burden of onchocerciasis and epilepsy in Mahenge following 25 years of CDTI in this region. Previous studies in Mahenge reported changes in onchocerciasis and epilepsy burden, showing persistent transmission of onchocerciasis and high prevalence and incidence of epilepsy despite two and half decades of CDTI [8,10,11,19,23–25]. Our findings are similar to those of the multicentric assessment of the seroprevalence of *O*. *volvulus* among people with epilepsy in onchocerciasis foci in the Democratic Republic of Congo, Cameroon, Tanzania (Mahenge), and Uganda, which reported prevalence ranging from 35.2% to 59.7% [15]. The observed prevalence of epilepsy and Ov16 among people with epilepsy in this study adds epidemiological evidence to the hypothesis that *O*. *volvulus* is associated with epilepsy, even though the pathophysiological mechanisms triggering seizures are still not well understood [16]. There are different hypotheses about the possible causes of the condition, including invasion of microfilariae into the central nervous system [26], deposition of pathogenic tau protein in the brain leading to neuronal dysfunction and neurodegenerative disease [27], and the cross-reactivity of Leomodin-1 antibodies with *O*. *volvulus* protein [28]. The evidence for any of these hypotheses is inconsistent between studies. In our study, the incidence based on the previous year was similar to the earlier reported figures in four villages in Mahenge in 2017/2018 [11]. Five-year incidence, also previously used, was slightly lower, potentially owing to more villages from varying altitudes with more intense onchocerciasis transmission compared to the 2017/2018 study that used data from only four villages [11]. The incidence of epilepsy in our study remains high compared to reports from other sub-Saharan countries such as Ghana, Ethiopia, and South Africa [29–31]. The epilepsy prevalence in Mahenge correlated with onchocerciasis exposure, with the villages located at medium altitudes having a high prevalence of onchocerciasis and epilepsy. The risk of acquiring onchocerciasis was eight times higher for the children residing in the villages at the medium altitudes than those at the lower and higher altitudes. An explanation for this could be that as rivers start to flow at higher altitudes, it is at medium altitudes that rapids occur, which are rich in oxygen and favour the breeding and survival of the *Simulium*. Hence, the high risk of transmission is in villages located at medium altitudes. This finding provides essential information about specific communities that may need additional control measures, particularly complemented vector control interventions [32], to accelerate the control of onchocerciasis and reduce epilepsy incidence.

The high prevalence of epilepsy in Ebuyu village may be due to variability of the altitude and environmental conditions in the western part of Ebuyu, which Isyaga village shares. Data from Ebuyu village shows that the prevalence of epilepsy in households located at altitudes ≥ 400 meters above sea level was 5.57%, while for those at lower altitudes, the prevalence was 2.8% (S1 Table). Conversely, the high prevalence in Ikungua village could be due to its location near the Luli River, which provides a conducive environment for *Simulium* to hide. The low Ov16 positivity rate in these villages could be due to low exposure to infective bites.

The observed higher prevalence of epilepsy in females may be related to activities that expose them to fast-flowing rivers or streams where they can be bitten by *Simulium* (for example, washing clothes or utensils). Additionally, male migration to other regions in search of employment, leaving females in the villages for extended periods, may account for the difference in prevalence between males and females, especially in the age group 30–39 years. Our findings differ from the study conducted in four villages of Mahenge in 2017, which showed no difference in the prevalence of epilepsy between males and females [11]. According to age categories, those aged 20 to 39 had the highest prevalence of epilepsy, possibly as a result of a shift in peak prevalence from the young individuals as a result of several years of CDTI implementation, in line with the results of other studies [11,33].

The overall prevalence of Ov16 in children aged 6–11 years was 11.8%, similar to the prevalences observed before the introduction of bi-annual CDTI in the area in 2019. This was to be expected, as most children were already infected before the switch to bi-annual CDTI. Switching from an annual to a biannual CDTI strategy reduced the incidence of epilepsy in four rural villages included in this study where this was assessed [25]. A similar finding was observed in Uganda and South Sudan onchocerciasis endemic areas [34,35]. In 2021, in Mahenge, the self-reported uptake of ivermectin was high, presumably because of OAE awareness campaigns by the NTD programme and the bi-annual distribution of ivermectin. In 2022, a study among school children in the Mahenge area in Mahenge B, Makanga, and Msogezi villages however, found that about half of the children eligible for ivermectin did not take the drug during the last round of MDA, which suggests over-reporting of ivermectin intake in our study, especially in households where there was only one person providing information for the entire household [24]. Sub-optimal uptake has been observed in some villages, which may impact the success of the onchocerciasis interruption and lengthen the time that ivermectin is distributed, and in turn, contribute to the persistence of onchocerciasis-associated epilepsy (S1 Table).

There was a high prevalence of Ov16 positivity among the people with epilepsy who did not receive ivermectin in the last distribution round compared to those who took it. Ivermectin, administered once or twice a year, has been shown to reduce the microfilaria load, relieve extreme itching, and prevent the progression toward blindness [23,36]. According to studies from other onchocerciasis-endemic countries, groups with low ivermectin uptake serve as a reservoir of infection and could develop morbidities associated with onchocerciasis, such as epilepsy [37–39].

Factors that increased the odds of developing epilepsy in Mahenge were being female; being older than ten years, being a resident of Mahenge for ≥20 years, especially in villages located at medium altitude, and being onchocerciasis positive. Being infected with onchocerciasis with a high microfilariae load could directly or indirectly trigger epilepsy. Similarly, being a resident of Mahenge for ≥20 years increases the risk of exposure to *Simulium* spp, resulting in onchocerciasis, which may later on trigger epilepsy. The age group of 20–39 years had higher odds of having epilepsy in Mahenge than other age groups. This finding is similar to other studies conducted in onchocerciasis endemic areas [13,40]. Studies have reported that before the introduction of CDTI, the risk of developing epilepsy was among children aged 10–20 years, but after CDTI initiation, a shift of the highest prevalence of epilepsy was observed in the 20–39 years age group [11,13,40].

### Study limitations

Our study has certain limitations. *O*. *volvulus* IgG4 antibodies were detected in the laboratory using the Ov16 rapid diagnostic test rather than the more sensitive Ov16 ELISA, which could impact the assessment of onchocerciasis seroprevalence. The use of the Ov16 ELISA could have resulted in more positive cases.

It should be noted that the bi-annual CDTI was only introduced three years before the survey. As a result, all children between the ages of 6–10 who tested positive for Ov16 had already been infected before the implementation of the bi-annual CDTI. Another limitation is that no entomological investigations were performed to identify blackfly breeding sites and infection rates.

Disclosure of epilepsy is a challenge in many communities, including Mahenge, owing to stigma [41], which may result in underreporting. People with epilepsy are at increased risk of dying due to seizure-related adverse events such as burns, status epilepticus, and drowning, which may affect the incidence rate reported.

The self-reported uptake of ivermectin could be subjected to recall bias because it requires recalling previous information, which could cause an over/underestimation of the prevalence of ivermectin uptake. The two-step approach used to identify people with epilepsy involved many interviewers, making it potentially susceptible to interviewer bias. This was, though, minimised by training the interviewers before and during data collection.

### Recommendations

The prevalence of onchocerciasis and epilepsy remains high in Mahenge despite using ivermectin for 25 years. We recommend strengthening the ongoing bi-annual ivermectin treatment, especially in the villages with a high prevalence of onchocerciasis and epilepsy. Formulating an appropriate ivermectin dose is needed for children <5 years since they acquire onchocerciasis before the recommended age for ivermectin uptake. This is an opportune time to introduce vector (*Simulium*) control interventions, especially in the villages 400–950 meters above sea level, to accelerate the fight against onchocerciasis further and prevent the potential future development of epilepsy.

## Supporting information

S1 Text Household Screening Questionnaire. (DOCX)

S2 Text Neurological Questionnaire. (DOCX)

S1 Acknowledgments Members of the EPInA Study Group. (DOCX)

S1 Table Table shows the study villages and distribution of median altitude, ivermectin coverage, and prevalence of Ov16 results and epilepsy. (DOCX)

S1 Data The data set used for analysis. (XLSX)
