# Supplementary material for: Prevalence of onchocerciasis and epilepsy in a Tanzanian region after a prolonged community-directed treatment with ivermectin
Source: PLoS Negl Trop Dis. 2024 Sep 6;18(9):e0012470. doi: 10.1371/journal.pntd.0012470 (PMC11410205; doi:10.1371/journal.pntd.0012470)
Supplement: S1 Text — (DOCX) [file pntd.0012470.s001.docx]

S1 File — Household screening questionnaire.

*NOTE: This questionnaire will be administered in a digitalized form on a tablet computer that will be used as a data collection tool during this survey. Within each household, all participants will be asked all the questions in sections 1.2 and 1.3 during one single interview sequence.*

DATE: _____ / _____ / ________
FULL NAME OF INTERVIEWER: _______________________________________________

HEALTH ZONE: _______________________________________________
HEALTH AREA: _______________________________________________

VILLAGE: _______________________________________________

**1.1. HOUSEHOLD CHARACTERISTICS**

UNIQUE HOUSEHOLD CODE:
 XX /XX /XX/XXXX (First 2 letters of District/Village, followed by number (e.g.0001))

GPS coordinates of household:
LATITUDE __ __. __ __ __ __ __LONGITUDE __ __. __ __ __ __ __ ALTITUDE _ __ __ __

Full name of household head:_______________________________________________
Mobile phone number of household head: ___________________________________
Ethnic group of household head: __________________________________________
Total number of people in the household: _____________________________________

Main income-generating activity of the family:  Farmer  Livestock breeder

Fisherman  Employee  Professional  Craftsman  other, specify:________

Has there been a family member who died from any form of epilepsy?
 YES NO DON’T KNOW

IF YES: When (year) ______ At what age: _____ years

IF YES: When (year) ______ At what age: _____ years

**1.2. INDIVIDUAL INTERVIEW WITH EACH HOUSEHOLD MEMBER**

Person ID: _______________________________________________
Full Name: _______________________________________________
Age: ___ years
Birth date: ___/___/____
Sex: Male Female

Is the person present during the interview visit? YES NO
Who is answering the questions? self mother household head other, specify _______________

**1.3. SCREENING FOR EPILEPSY CASES**

*If at least one of the 5 questions is answered YES, the electronic questionnaire will automatically report the person for an invitation to participate in the neurological survey for case verification.*

**QUESTION 1.**Have you ever lost consciousness and experienced:

1. Loss of bladder control? YES NO DON’T KNOW

b) Foam at the mouth? YES NO DON’T KNOW

**QUESTION 2.**Have you ever experienced absence(s) or sudden loss(es) of contact with the surroundings, for a short duration of time?

YES NO DON’T KNOW

**QUESTION 3.**Have you ever experienced sudden, uncontrollable twitching or shaking of your arms, legs, or head, for a period of a few minutes?

YES NO DON’T KNOW

**QUESTION 4.**
Do you sometimes experience sudden and brief bodily sensations, see or hear things that are not there, or smell strange odors?

YES NO DON’T KNOW

**QUESTION 5.**Have you ever been told that you are suffering from epilepsy or that you have already had epileptic fits?

YES NO DON’T KNOW

**1.3. IVERMECTIN USE**

Have you been taking ivermectin during the last distribution?

YES NO DON’T KNOW
