# Supplementary material for: Prevalence of onchocerciasis and epilepsy in a Tanzanian region after a prolonged community-directed treatment with ivermectin
Source: PLoS Negl Trop Dis. 2024 Sep 6;18(9):e0012470. doi: 10.1371/journal.pntd.0012470 (PMC11410205; doi:10.1371/journal.pntd.0012470)
Supplement: S1 Acknowledgments — (DOCX) [file pntd.0012470.s003.docx]

**EPInA Study Group**

Albert Akpalu, Anthony Godi, Arjune Sen, Bruno P. Mmbando, Charles R. Newton, Cynthia Sottie, Damazo T. Kadengye, Dan Bhwana, Daniel Mtai Mwanga, Daniel Nana Yaw Abankwah, David McDaid, Dorcas Muli, Emmanuel Darkwa, Frederick Murunga Wekesah, Gergana Manolova, Gershim Asiki, Harieth Hyera, Helen Cross, Henrika Kimambo, Isolide S. Massawe, Josemir Sander, Mary Bitta, Mercy Atieno, Neerja Chowdhary, Patrick Adjei, Pendo Faustine, Peter Otieno, Richard Walker, Ryan Wagner, Sabina Asiamah, Samuel Iddi, Simone Grassi, Sloan Mahone, Sonia Vallentin, Stella Waruingi, Symon Kariuki, Tarun Dua, Thomas Kwasa, Timothy Denison, Vivian Mushi and William Matuja
