# Supplementary material for: Prevalence of onchocerciasis and epilepsy in a Tanzanian region after a prolonged community-directed treatment with ivermectin
Source: PLoS Negl Trop Dis. 2024 Sep 6;18(9):e0012470. doi: 10.1371/journal.pntd.0012470 (PMC11410205; doi:10.1371/journal.pntd.0012470)
Supplement: S1 Table — (DOCX) [file pntd.0012470.s004.docx]

| **S1 Table. Study villages and distribution of median altitude, ivermectin coverage, and prevalence of Ov16 results and epilepsy** | | | | | | | | | | | | | | | | |
| --- | --- | --- | --- | --- | --- | --- | --- | --- | --- | --- | --- | --- | --- | --- | --- | --- |
|  |  | **Median altitude, (range)** |  | **Ivermectin (>5 years)** | |  | **OV16 test (5-11 years)** | |  | **Epilepsy (all ages)** | |  |  |  |  |  |
| **Strata** | **Village** | **(mASL)** |  | **N** | **Used (%)** |  | **N** | **OV16 +ve** |  | **N** | **Yes** |  |  |  |  |  |
| Low | Iragua | 306 (202 - 472) |  | 1,861 | 1,382 (74.2) |  | 237 | 2 (0.8) |  | 2,359 | 39 (16.5) |  |  |  |  |  |
|  | Kichangani | 311 (210 - 463) |  | 2,457 | 2,204 (89.7) |  | 223 | 2 (0.9) |  | 2,912 | 28 (9.6) |  |  |  |  |  |
|  | Lukande | 320 (215 - 382) |  | 1,281 | 998 (77.9) |  | 91 | 18 (19.8) |  | 1,596 | 23 (14.4) |  |  |  |  |  |
|  | Ikungua | 322 (259 - 455) |  | 429 | 368 (85.7) |  | 37 | 2 (5.4) |  | 508 | 12 (23.6) |  |  |  |  |  |
|  | Magereza | 326 (178 - 489) |  | 3,184 | 2,159 (67.8) |  | 196 | 3 (1.5) |  | 3,896 | 56 (14.4) |  |  |  |  |  |
|  | Idunda | 331 (269 - 383) |  | 1,368 | 1,265 (92.4) |  | 98 | 7 (7.1) |  | 1,567 | 33 (21.1) |  |  |  |  |  |
|  | Euga | 359 (275 - 503) |  | 1,408 | 1298 (92.1) |  | 123 | 2 (1.6) |  | 1,581 | 17 (10.8) |  |  |  |  |  |
|  | Gombe | 360 (216 - 430) |  | 450 | 402 (89.3) |  | 36 | 2 (5.6) |  | 536 | 11 (20.5) |  |  |  |  |  |
|  | Chirombola | 367 (305 - 438) |  | 2,205 | 2079 (94.2) |  | 168 | 0 (0) |  | 2,506 | 30 (12.0) |  |  |  |  |  |
|  | Ebuyu | 381 (256 - 439) |  | 1,828 | 1649 (90.2) |  | 192 | 5 (2.6) |  | 2,088 | 68 (32.6) |  |  |  |  |  |
|  | Mtukula | 381 (326 - 549) |  | 1,143 | 1,065 (93.1) |  | 72 | 4 (5.6) |  | 1,311 | 16 (12.2) |  |  |  |  |  |
| Medium | Chikuti | 401 (192 - 775) |  | 1,020 | 899 (88.1) |  | 58 | 12 (20.7) |  | 1,206 | 33 (27.4) |  |  |  |  |  |
|  | Mzelezi | 401 (316 - 599) |  | 1,910 | 1,765 (92.4) |  | 106 | 12 (11.3) |  | 2,286 | 51 (22.3) |  |  |  |  |  |
|  | Kituti | 416 (358 - 576) |  | 1,373 | 1,270 (92.4) |  | 172 | 15 (8.7) |  | 1,592 | 37 (23.2) |  |  |  |  |  |
|  | Isyaga | 428 (367 - 665) |  | 1,755 | 1,651 (94.0) |  | 152 | 15 (9.9) |  | 2,031 | 63 (31.0) |  |  |  |  |  |
|  | Mgolo | 459 (294 - 539) |  | 1,156 | 1,103 (95.4) |  | 86 | 14 (16.3) |  | 1,312 | 48 (36.6) |  |  |  |  |  |
|  | Lyandu | 480 (409 - 576) |  | 497 | 411 (82.6) |  | 55 | 23 (41.8) |  | 581 | 19 (32.7) |  |  |  |  |  |
|  | Isaka | 506 (392 - 950) |  | 656 | 569 (86.7) |  | 49 | 28 (57.1) |  | 756 | 36 (47.6) |  |  |  |  |  |
|  | Mdindo | 532 (456 - 791) |  | 643 | 597 (92.8) |  | 61 | 19 (31.1) |  | 874 | 33 (37.8) |  |  |  |  |  |
|  | Msogezi | 553 (460 - 731) |  | 1,617 | 1,501 (92.8) |  | 92 | 19 (20.7) |  | 1,985 | 53 (26.7) |  |  |  |  |  |
|  | Majengo | 608 (211 - 829) |  | 791 | 750 (94.8) |  | 57 | 28 (49.1) |  | 929 | 30 (32.3) |  |  |  |  |  |
|  | Kisewe | 653 (560 - 868) |  | 860 | 660 (76.7) |  | 92 | 32 (34.8) |  | 1,008 | 40 (39.7) |  |  |  |  |  |
|  | Namgezi | 734 (365 - 848) |  | 706 | 676 (95.7) |  | 16 | 7 (43.8) |  | 805 | 30 (37.3) |  |  |  |  |  |
|  | Makanga | 833 (663 - 1116) | | 1,724 | 1,565 (90.7) |  | 171 | 30 (17.5) |  | 2,012 | 73 (36.3) |  |  |  |  |  |
|  | Sali | 879 (419 - 981) |  | 1,207 | 1,109 (91.8) |  | 72 | 26 (36.1) |  | 1,467 | 44 (30.0) |  |  |  |  |  |
| High | Isongo | 924 (547 - 1076) | | 2,440 | 2,235 (91.5) |  | 268 | 46 (17.2) |  | 2,836 | 80 (28.2) |  |  |  |  |  |
|  | Epanko | 959 (339 - 1135) | | 1,430 | 1291 (90.2) |  | 103 | 11 (10.7) |  | 1,657 | 13 (7.8) |  |  |  |  |  |
|  | Safari Road | 959 (892 - 1050) | | 2,147 | 1,833 (85.3) |  | 122 | 7 (5.7) |  | 2,551 | 32 (12.5) |  |  |  |  |  |
|  | Uponera | 964 (604 - 1125) | | 1,786 | 1,632 (91.3) |  | 74 | 12 (16.2) |  | 2,103 | 35 (16.6) |  |  |  |  |  |
|  | Mbagula | 968 (852 - 1218) | | 994 | 897 (90.2) |  | 83 | 10 (12) |  | 1,120 | 21 (18.8) |  |  |  |  |  |
|  | Mawasiliano | 1031 (798 - 1120) | | 1,313 | 1,127 (85.8) |  | 64 | 5 (7.8) |  | 1,539 | 19 (12.3) |  |  |  |  |  |
|  | Matumbala | 1039 (974 - 1111) | | 1,180 | 1,110 (94.0) |  | 94 | 4 (4.3) |  | 1,335 | 18 (13.5) |  |  |  |  |  |
|  | Vigoi | 1062 (964 - 1291) | | 1,489 | 1,374 (92.2) |  | 106 | 8 (7.5) |  | 1,698 | 22 (13.0) |  |  |  |  |  |
|  | Nawenge | 1092 (787 - 1203) | | 1,779 | 1,598 (89.8) |  | 150 | 17 (11.3) |  | 2,061 | 31 (15.0) |  |  |  |  |  |
|  | Total | 455 (178 - 1291) | | 48,087 | 42,492 (88.4) |  | 3776 | 447 (11.8) |  | 56,604 | 1194 (21.1) |  |  |  |  |  |
